# How dietary amino acids and high protein diets influence insulin secretion

**DOI:** 10.14814/phy2.15577

**Published:** 2023-01-25

**Authors:** Yuuki Yanagisawa

**Affiliations:** ^1^ Department of Metabolism, Digestion and Reproduction Imperial College London London UK

**Keywords:** dietary amino acids, glucose homeostasis, insulinotropic activity, satiety

## Abstract

Glucose homeostasis is the maintenance and regulation of blood glucose concentration within a tight physiological range, essential for the functioning of most tissues and organs. This is primarily achieved by pancreatic secretion of insulin and glucagon. Deficient pancreatic endocrine function, coupled with or without peripheral insulin resistance leads to prolonged hyperglycemia with chronic impairment of glucose homeostasis, most commonly seen in diabetes mellitus. High protein diets (HPDs) are thought to modulate glucose homeostasis through various metabolic pathways. Insulin secretion can be directly modulated by the amino acid products of protein digestion, which activate nutrient receptors and nutrient transporters expressed by the endocrine pancreas. Insulin secretion can also be modulated indirectly, through incretin release from enteroendocrine cells, and via vagal neuronal pathways. Additionally, glucose homeostasis can be promoted by the satiating effects of anorectic hormones released following HPD consumption. This review summarizes the insulinotropic mechanisms by which amino acids and HPDs may influence glucose homeostasis, with a particular focus on their applicability in the management of Type 2 diabetes mellitus.

## Graphical abstract

How dietary amino acids influence insulin secretion. Dietary amino acids are sensed by nutrient sensors expressed by pancreatic alpha and beta cells, and by enteroendocrine cells. This leads to the secretion of insulin, glucagon and incretin hormones respectively. BioRender, 2022.

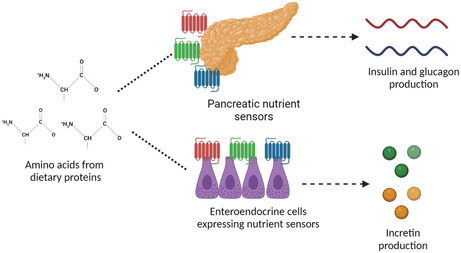

## 1 INTRODUCTION

Glucose is the simplest and most abundant monosaccharide known to man and is the primary biological fuel source for the human body (Hantzidiamantis & Lappin, 2022). Glucose is obtained from three fundamental sources—exogenously from dietary intake, from glycogenolysis in the liver and muscle, and gluconeogenesis in the liver, kidneys, and intestine (Aronoff et al., 2004).

Glucose homeostasis is the maintenance and regulation of blood glucose levels within the narrow range of 4 to 6 mmol/L and is critical for maintaining the normal physiological functioning of most organs and tissues (Pozo, 2018; Roder et al., 2016). Glucose homeostasis is achieved through the coordination of glucose sensing mechanisms which detect fluctuations in blood glucose concentration with multiple effector responses which act to re‐establish blood glucose levels within this tight target range (Pozo, 2018). Effector responses are mediated by several complex neural and endocrine networks, involving several neurotransmitters, neuropeptides, and glucoregulatory hormones (Aronoff et al., 2004). The pancreas is a critical source of glucoregulatory hormones, which are synthesized and secreted by collections of specialized endocrine cells found within the islets of Langerhans (Aronoff et al., 2004; Petersen, 2018; Roder et al., 2016). Insulin is an anabolic hormone secreted by pancreatic β cells in response to increased blood glucose concentration (Petersen, 2018). Insulin decreases blood glucose concentrations by promoting glucose uptake into muscle and adipose tissues, stimulating hepatic glycogenesis, and downregulating gluconeogenesis (Aronoff et al., 2004; Petersen, 2018). Insulin also increases cellular uptake of amino acids from the peripheral circulation, facilitating intracellular protein synthesis (Zakaria et al., 2021). In contrast, glucagon is released from α cells in states of fasting and hypoglycemia. Glucagon raises blood glucose concentration by inducing gluconeogenesis and glycogenolysis (Roder et al., 2016).

In addition to insulin and glucagon, several other glucoregulatory hormones contribute to glucose homeostasis (Aronoff et al., 2004; Renner et al., 2015). These include somatostatin, and the incretins glucagon‐like peptide 1 (GLP‐1) and glucose‐dependent insulinotropic peptide (GIP). Somatostatin is secreted from delta cells within the islets of Langerhans, stomach, and small intestine (O'Toole & Sharma, 2022). Somatostatin inhibits the release of both insulin and glucagon, as well as the release of pancreatic exocrine secretions (O'Toole & Sharma, 2022; Strowski et al., 2000). In comparison, the latter two peptide hormones GLP‐1 and GIP are secreted from L‐ and K‐enteroendocrine cells of the intestine, respectively (Aronoff et al., 2004). Both GLP‐1 and GIP potentiate glucose‐dependent insulin secretion, enhance β‐cell proliferation, and downregulate β‐cell apoptosis, and GLP‐1 also inhibits glucagon secretion (Renner et al., 2015).

Chronic impairment or deficiency of these glucoregulatory responses can lead to the development of diabetes mellitus, a major global health epidemic associated with considerable clinical sequelae, and vast financial and healthcare ramifications (O'Connell & Manson, 2019). At present, 463 million people worldwide are estimated to suffer from diabetes mellitus, which can be classified as Type 1 or Type 2 diabetes mellitus (T1DM or T2DM) (Saeedi et al., 2019). In the UK, approximately 90% of the diabetic population have T2DM, with T1DM accounting for 8% of patients (Diabetes UK, n.d.). T2DM is characterized by peripheral resistance to insulin coupled with progressive β‐cell failure and relative insulin deficiency (Strowski et al., 2000). In contrast, T1DM is defined by complete or near complete insulin deficiency due to the autoimmune destruction of β cells (Diabetes UK, n.d.).

Given the long‐term complications of T2DM such as cardiovascular and cerebrovascular disease, congestive heart failure, and predisposition to recurrent infection, an ever‐growing demand exists to better understand and improve persistent hyperglycemia associated with T2DM, potentially through the use of high protein diets (HPDs) (Diabetes UK, n.d.; Toft‐Nielsen et al., 2001).

A HPD is specifically defined as a diet supplying 35% or more of daily total energy intake in the form of protein (O'Connell & Manson, 2019; Saeedi et al., 2019). Proteins are macronutrients, composed of one or more chains of different amino acids. Animal proteins such as whey, casein, egg, fish, beef, and chicken, along with plant‐based proteins including soy, pea, brown rice, and chickpea are essential for multiple physiological and homeostatic activities within the body (Lupton et al., 2005). In particular, amino acids and HPDs can also influence glucose homeostasis (Galbreath et al., 2018; Lupton et al., 2005; Morell, 2017; Parker et al., 2002; Pasiakos et al., 2013; Stokes et al., 2018; Tipton, 2011). Multiple studies conducted in rodents and humans have shown that consumption of protein and amino acids leads to weight loss and improves glucose homeostasis (Galbreath et al., 2018; Morell, 2017; Parker et al., 2002; Pasiakos et al., 2013; Stokes et al., 2018). Although the effects of HPDs are generated by the physiological activities of their constituent amino acids, pharmacological administration of amino acids can differentially impact glucose homeostasis. This is in part due to differences in digestion time, the rate of gastric emptying and release into the blood stream, and the utilization following digestion (Pena et al., 2015).

Despite the physiological benefits of amino acids and HPDs, there are concerns regarding the implications of their long‐term consumption, particularly nephrotoxicity (Cirillo et al., 2014; Knight et al., 2003; Pasiakos et al., 2013). Several long‐term observational studies conducted in adults have found an association between high protein intake and decline in renal function, in both participants with normal renal function and those with pre‐existing renal disease (Cirillo et al., 2014; Knight et al., 2003). These complications, along with relatively poor levels of adherence to HPDs, have raised the question if the mechanisms by which amino acids and HPDs improve glucose homeostasis can be exploited without the need for the consumption of high levels of dietary protein.

This review will examine the different insulinotropic and non‐insulinotropic mechanisms by which amino acids and HPDs influence glucose homeostasis and their supporting evidence. The translational potential to exploit these mechanisms for the management of diabetes mellitus will also be discussed.

## 2 INSULINOTROPIC EFFECTS OF HIGH PROTEIN DIETS AND DIETARY AMINO ACIDS

### 2.1 Direct insulinotropic mechanisms

Improved glycemic control observed with consumption of HPDs is most commonly attributed to the putative insulinotropic activity of amino acids (Galbreath et al., 2018; Lupton et al., 2005; Morell, 2017; Parker et al., 2002; Pasiakos et al., 2013; Stokes et al., 2018; Tipton, 2011). This can be achieved by amino acids directly modulating pancreatic β cell, and to a lesser extent, α‐cell function. There are several mechanisms which may account for this. The first and most established mechanism is known as the “Triggering pathway” (Cirillo et al., 2014; Parker et al., 2002; Pena et al., 2015). The triggering pathway is comprised of several events, including adenosine triphosphate generation (ATP) from the tricarboxylic acid cycle, which requires oxidized amino acid substrates. ATP is responsible for closure of ATP‐sensitive potassium channels, causing β‐cell membrane depolarization (McClenaghan et al., 1996; Newsholme et al., 2006). Cationic amino acids such as arginine and lysine are responsible for enhancing β‐cell depolarization (Henquin, 2000; Liu et al., 2008; McClenaghan et al., 1996). Additionally, co‐transportation of l‐alanine and proline with sodium ions is also thought to contribute to β‐cell depolarization (Dunne et al., 1990). Depolarization leads to calcium channel activation, with intracellular calcium influx ultimately resulting in insulin exocytosis (Lu & Li, 2018). Evidence suggests that glutamate may control calcium‐induced insulin exocytosis (Henquin, 2000; Maechler & Wollheim, 1999).

The role of the mammalian target of rapamycin complex 1 (mTORC1), a protein kinase signaling complex, is also key in amino acid‐mediated insulin secretion (Conigrave et al., 2000; Kwon et al., 2004). mTOR is responsible for the regulation of protein synthesis, specifically insulin synthesis in the β cell, by orchestrating downstream mRNA translation and increasing the capacity of ribosomal machinery (Conigrave et al., 2000; Kwon et al., 2004; Newsholme et al., 2006). Amino acids are one of the key ligands responsible for activation of mTORC1 in β cells, which is dependent on Ras‐related guanosine triphosphate‐binding guanosine triphosphatases (Rag GTPases) (Takahara et al., 2020). A total of 4 Rag proteins exist (Rag A, B, C and D), which in their resting state, are tethered to the membranes of lysosomes, due to their association with the pentameric Ragulator complex (Nellist et al., 2003). Intracellular uptake of amino acids into β‐cells promotes a shift of Rag GTPases to their active nucleotide‐bound state, to either GTP guanosine diphosphate (GDP) (Takahara et al., 2020). Activated Rag GTPases are able to bind to the regulatory‐associated protein of mTOR (Raptor, one of the key components of mTORC1), which recruits mTORC1 to the lysosomal surface. At the lysosomal surface mTORC1 is ultimately activated by interacting with the small GTPase Ras homolog enriched in brain (Rheb) at the lysosomal surface (Nellist et al., 2003; Takahara et al., 2020).

Further work by Zhang et al. (2021) in mice has also shown that the GTPase protein Rab1A located on the membranes of the endoplasmic reticulum and Golgi apparatus is required for amino acid‐mediated mTORC1 activation and insulin secretion (Abraham, 2015). Zhang et al. (2021) studied the effects of treatment with Rab1A versus small inhibiting RNA for Rab1A (siRNA) on amino acid‐mediated activation of mTORC1 and insulin expression in MIN6 β‐cells. MIN6 β cells treated with Rab1A in vitro exhibited a significant increase in insulin mRNA expression versus MIN6 cells treated with siRNA. The addition of an amino acid substrate further enhanced the increase in insulin expression in MIN6 cells treated with Rab1A, as did the individual addition of the Rapamycin substrate, demonstrating that amino acid‐mediated activation of mTORC1 is dependent on the Rab1A GTPase.

Zhang et al. (2021) also studied the effect of generated tamoxifen‐induced whole‐body (global) Rab1A knockout adult mice. In comparison with wild‐type mice, Rab1A knockout mice were found to have significantly reduced insulin blood levels, hyperglycemia and impaired glucose tolerance.

In addition to amino acid‐mediated activation of mTORC1, mTORC1 signaling is also known to be modulated by chemical signals such as calcium (Li et al., 2016; Newsholme et al., 2006). Li et al. (2016) showed that mTORC1 is achieved through lysosomal calcium release through the transient receptor potential channel 1 (TRPML1). Human embryonic kidney 293 cells (HEK293 cells) were treated with short hairpin RNA (shRNA) targeting TRPML1 (to achieve knockdown) or control vehicle. Treatment with shRNA resulted in a depletion of mTORC1 versus treatment with control vehicle. Following lysosomal release, calcium specifically activates mTORC1 by inducing association of calmodulin with mTOR. Treatment of human umbilical vein endothelial cells (HUVECs) with the calmodulin antagonist significantly inhibited mTORC1 activity versus treatment with positive and negative control vehicles.

In order for amino acids to mediate insulin and glucagon secretion they must first be sensed (Cork, 2018; Gribble & Reimann, 2019; Gutierrez‐Aguilar & Woods, 2011; Koepsell, 2020; Newsholme et al., 2010). Nutrient sensors are molecular and cellular machines responsible for detecting amino acids in addition to other nutrient components such as glucose, fatty acids, minerals, and anions (Bohorquez et al., 2015; Newsholme et al., 2010). These include the G‐protein‐coupled receptor family C, group 6, subtype A (GPRC6A), the G‐protein coupled receptor 142 (GPR142), the Umami Taste receptor type 1 member 1/ member 3 (Umami T1R1/T1R3), several free fatty acid receptors (Free fatty acid receptor 1 and GPR119) and the calcium‐sensing receptor (CaSR) (Cork, 2018; Gribble & Reimann, 2019; Gutierrez‐Aguilar & Woods, 2011; Koepsell, 2020; Newsholme et al., 2010). As well as being expressed by α and β cells, nutrient sensors are expressed by enteroendocrine cells (EECs) and vagal afferent neurons of the enteroendocrine system and parasympathetic nervous system respectively (Bohorquez et al., 2015; Newsholme et al., 2010).

### 2.2 Indirect insulinotropic mechanisms

The enteroendocrine system and parasympathetic nervous system are complex pathways within the gut–brain axis which ultimately influence insulin, and to a lesser extent, glucagon secretion (Gribble & Reimann, 2019; Gutierrez‐Aguilar & Woods, 2011; Lu & Li, 2018; Newsholme et al., 2010).

The enteroendocrine system is composed of EECs which are found in the gut epithelium from the stomach to the colon (Gutierrez‐Aguilar & Woods, 2011). EECs can be classed as either “open,” with microvilli expressed on the apical membrane extending to the gut lumen, or “closed” with microvilli which do not reach the lumen. The basolateral surface of EECs faces the interstitial space (Gutierrez‐Aguilar & Woods, 2011; Koepsell, 2020). The apical membranes of EECs express multiple chemoreceptors and nutrient sensors previously described (Cork, 2018). Following nutrient sensing, EECs have the ability to directly connect and communicate with afferent and efferent neurons through neuroepithelial circuits (Bohorquez et al., 2015). Neuropods, the cytoplasmic process of EECs is directly penetrated by nerves, which allows for synaptic communication from the EEC to neurons, relaying information of nutrient status. EECs are also thought to act in a paracrine fashion, modulating other EECs (Bertrand, 2009; Cummings, 2007).

Peptide hormones are implicated in multiple different physiological responses, of which glucose‐dependent insulinotropic peptide (GIP) and glucagon‐like peptide 1 (GLP‐1) are the most integral in glucose homeostasis (Koepsell, 2020). Secretion of GIP and GLP‐1 is mediated by K‐EECs found predominantly in the duodenum, and L‐EECs, found predominantly in the ileum and colon, respectively. GIP and GLP‐1 are both classified as incretin peptide hormones, which promote insulin secretion and dampen glucagon release, improving glucose tolerance in response to orally ingested nutrients (Gutierrez‐Aguilar & Woods, 2011; Koepsell, 2020). Additionally, GLP‐1 amplifies β‐cell proliferation and survival, while attenuating β‐cell apoptosis (Cork, 2018).

Amino acids may also co‐ordinate insulin and glucagon secretion via the parasympathetic nervous system, specifically the vagus nerve. The vagus nerve is responsible for regulating physiological activities within the cardiovascular, respiratory, and renal systems, as well as pancreatic endocrine function and glucose homeostasis (Bai et al., 2019). With respect to glucose homeostasis, the vagus nerve facilitates bidirectional communication between the gut and the brain via its afferent and efferent branches (Brookes et al., 2013). Vagal afferent neurons densely innervate the lamina propria of the gut mucosa, transmitting sensory information regarding nutrient and peptide hormone concentrations to the dorsal vagal complex of the brainstem (Bai et al., 2019; Brookes et al., 2013). The vagal efferent branch then conveys motor information to the endocrine pancreas, dictating insulin and glucagon secretion. Although several amino acid sensors including CaSR and GPR35 are known to be expressed by vagal afferent neurons, there has been no definitive evidence at present that dietary amino acids modulate nutrient sensor‐expressing vagal afferent fibers to influence insulin secretion and glucose homeostasis (Egerod et al., 2018).

## 3 THE EFFECTS OF DIFFERENT HIGH PROTEIN DIETS ON GLUCOSE HOMEOSTASIS

To date there have been numerous studies on how different HPDs influence glucose homeostasis, insulin secretion, weight loss and future risk of T2DM, both in healthy individuals and those with obesity and/or T2DM (Rietman, Schwarz, Tome, et al., 2014). HPDs are classified according to duration and energy restriction, with respect to daily calorie requirements for adult males and females (Das et al., 2010).

### 3.1 Short term, energy‐balanced high protein diets

The impact of short term, energy‐balanced HPDs in healthy subjects (BMI < 25 kg/m^2^) has unfortunately failed to demonstrate any significant change glucose homeostasis and insulin secretion (Rietman, Schwarz, Blokker, et al., 2014; Warland et al., 2008). For example, Warland et al. (2008) studied how a 10‐day HPD (3 g protein/kg of fat‐free mass/day) influenced insulin sensitivity in healthy adults in a randomized crossover trial. In comparison with a “usual protein intake” diet of 1.5 g protein/kg of fat‐free mass/day, no significant difference was found in insulin sensitivity. This suggests that insulin secretion is not acutely modulated by HPDs in individuals with normal pancreatic endocrine function. These findings were also mirrored by Rietman, Schwarz, Blokker, et al. (2014).

The impact of short term, energy‐balanced HPDs on glucose and insulin homeostasis in overweight and obese subjects has been shown to augment insulin sensitivity, but not overall glucose homeostasis. Pal et al. (2010) found a significant decrease in fasting insulin levels and insulin resistance with a HPD supplemented with whey protein (Pal et al., 2010). Seventy non‐diabetic, overweight, and obese individuals (BMI of 25 to 40 kg/m^2^) were randomized to either a whey protein‐supplemented HPD or a glucose‐supplemented control diet, which was consumed twice daily over a period of 12 weeks. After study completion, a significant reduction in fasting insulin levels and insulin resistance was observed in the whey protein group versus glucose control diet.

### 3.2 Short term, energy‐restricted high protein diets

The impact of short term, energy‐restricted HPDs on glucose homeostasis has predominantly been studied in overweight and obese subjects. Tettamanzi et al. (2021) conducted a randomized controlled crossover trial of a HPD or Mediterranean control diet in 16 non‐diabetic, obese female subjects over 3 weeks. Each diet had 500 calories less than the average female daily calorie requirement. After study completion, a significant reduction in insulin resistance and fasting blood glucose was observed in the HPD group. The attenuation in insulin resistance and enhancement in glucose homeostasis was not influenced by weight loss, as there was no significant change in body weight between the 2 groups.

Despite the findings of Tettamanzi et al. (2021), other studies by Abete et al. (2009) and Ratliff et al. (2009) determined that short term, energy‐balanced HPDs only reduce insulin resistance and enhance glucose homeostasis in the presence of statistically significant weight loss in non‐diabetic overweight and obese subjects.

### 3.3 Long‐term high protein diets

In comparison with short‐term HPDs, long‐term HPDs >6 months in duration have been shown to increase insulin resistance and lead to glucose intolerance in healthy subjects and increase the long‐term risk of developing T2DM (Sluijs et al., 2010; Song et al., 2004). This was highlighted by two large prospective cohort studies conducted in healthy adults over 8 and 10 years by Song et al. (2004) and Sluijs et al. (2010), respectively.

It has been hypothesized that hyperactivation of mTORC1 is responsible for the increased risk of insulin resistance and T2DM observed with long‐term HPDs (El Hiani et al., 2018). The presence of amino acid overload is thought to lead to reduced insulin receptor substrate degradation and reduced protein kinase B (Akt)‐ Akt substrate of 160 KDa (AS160) activity, which ultimately impairs insulin‐stimulated glucose uptake (El Hiani et al., 2018).

In overweight and obese subjects, however, long‐term HPDs have been found to attenuate insulin resistance and improve overall glucose homeostasis (Drummen et al., 2018; Marco‐Benedi et al., 2020; Mateo‐Gallego et al., 2017). For example, Drummen et al. (2018) showed that a 2‐year HPD in 25 overweight and obese non‐diabetic subjects led to a significant decline in insulin resistance, and fasting insulin and glucose concentrations versus moderate protein control diet. The enhancement in glucose homeostasis was accompanied by a significant reduction in body weight, which is likely to be at least partially responsible for the observed metabolic changes.

Studies by Marco‐Benedi et al. (2020) and Mateo‐Gallego et al. (2017) have also concluded similar findings.

**Table:** 

High protein diet type	Metabolic effects	
	Healthy subjects	Obese subjects
Short‐term (1 week to 6 months), energy balanced, high protein diet	No significant change in serum insulin levels in healthy individuals (Rietman, Schwarz, Blokker, et al., 2014; Warland et al., 2008)	Reduced insulin resistance in overweight and obese subjects (Pal et al., 2010)
Short‐term (1 week to 6 months, energy restricted, high protein diet)	Lack of studies available	Reduced insulin resistance, decrease in serum insulin levels (Tettamanzi et al., 2021) Weight loss, decrease in fasting blood glucose concentration, reduction in insulin resistance, decrease in serum insulin levels (Abete et al., 2009; Ratliff et al., 2009)
Long‐term high protein diet (>6 months)	Increased insulin resistance, glucose intolerance (Sluijs et al., 2010; Song et al., 2004)	Reduced insulin resistance (Drummen et al., 2018; Marco‐Benedi et al., 2020; Mateo‐Gallego et al., 2017)

### 3.4 Branched chain amino acids

Branched chain amino acids (BCAAs) are a prominent group of amino acids which constitute a major source of dietary protein (Rietman, Schwarz, Tome, et al., 2014). There are 3 BCAAs‐ leucine, isoleucine and valine which are essential amino acids (Layman, 2004). The beneficial impact of BCAAs on glucose homeostasis remains controversial (Chen et al., 2016; Ding et al., 2021; Labonte et al., 2017; Lynch, 2014; Newgard et al., 2009; Nilsson et al., 2007). In the short term, consumption of BCAAs has been shown to augment glucose homeostasis through enhancing insulin secretion and reduce postprandial glucose concentrations (Ding et al., 2021; Nilsson et al., 2007). These metabolic effects are perhaps unsurprising, given that BCAAs play a key role in β‐cell signaling and metabolism through activation of mTORC1 (Xie & Herbert, 2011).

In the long term, however, persistently elevated concentrations of BCAAs have been associated with insulin resistance, obesity and have actually been used as a risk marker for T2DM (Chen et al., 2016; Labonte et al., 2017; Lynch, 2014; Newgard et al., 2009; Xie & Herbert, 2011). Newgard et al. (2009) identified a linear relationship between increasing levels of BCAAs and insulin resistance. This is thought to be the result of several factors. Chronic hyperactivation of mTORC1 blunting insulin/growth factor signaling which eventually leads to reduced insulin sensitivity as well as BCAAs downregulating insulin clearance (Lee et al., 2016). Ultimately, this is thought to result β‐cell exhaustion (Wang et al., 2011).

### 3.5 Nutrient sensors

#### 3.5.1 The calcium sensing receptor (CaSR)

The CaSR is perhaps the best‐studied nutrient sensor with respect to amino acid detection. The CaSR is a pleiotropic receptor which has several physiological functions (Thakker, 2012). It is expressed in calciotropic organs such as the parathyroid glands, kidneys, and skeletal system, where it regulates extracellular calcium homeostasis and bone metabolism, as well as being expressed in non‐calciotropic tissues including pancreatic α and β cells, EECs, vagal afferent neurons of the peripheral nervous system, and the brain (Egerod et al., 2018; Thakker, 2012). Its expression in non‐calciotropic tissues in particular allows the CaSR to influence amino acid sensing, and secretion of peptide hormones, insulin, and glucagon (Hannan et al., 2018). The CaSR is therefore known to have a strong affinity for aromatic amino acids such as l‐phenylalanine (l‐phe), which is abundant in most HPDs (Hannan et al., 2018).

The ability of the CaSR to influence insulin secretion has been illustrated by several groups. Gray et al. (2006) studied the effect of incubating isolated human pancreatic islets ex vivo with or without the CaSR agonist and calcimimetic R‐568, with varying concentrations of calcium. Incubation with R‐568 in the absence of calcium caused a small but significant rise in insulin secretion, in comparison with islets incubated in glucose alone. In contrast, islets incubated with R‐568 and increasing concentrations of calcium exhibited profound increases in insulin secretion. Although this supports the role of the CaSR in mediating insulin secretion, it highlights the need for calcium itself in addition to a calcimimetic in order to fully modulate the CaSR and evoke greater insulin responses. This is supported by Conigrave et al. (2000) who demonstrated that in order for l‐amino acids such as l‐phe and l‐tryptophan (l‐trp) to modulate the CaSR, a threshold level of extracellular calcium is required. It is hypothesized that amino acids may act as positive allosteric modulators of the CaSR, with a stereo‐selective response to their l‐isomers in particular, increasing sensitivity to extracellular calcium and thus lowering the threshold for CaSR activation (Nemeth et al., 1998).

More recent work by Alamshah et al. (2017) has demonstrated that l‐phe enhances glucose homeostasis in vivo by stimulating insulin release, which is potentially achieved through modulation of the pancreatic CaSR. Oral gavage of l‐phe resulted in lower plasma glucose concentrations and significantly greater plasma insulin levels in rats, potentially by activating CaSR‐expressing β‐cells.

The ability of l‐phe to stimulate insulin and glucagon release and enhance glycemic control has also been replicated in humans. Amin et al. (2020) showed that consumption of l‐phe in healthy humans lead to a significant increase in insulin and glucagon release combined with reduced postprandial glycemia versus placebo. These findings have also been replicated by Fitzgerald et al. (2020) and Nuttall et al. (2006) in healthy humans.

The CaSR is also thought to regulate incretin hormone release at the level of K‐ and L‐EECs. Mace et al. (2012) studied GIP and GLP‐1 release from K‐ and L‐EECs respectively in isolated loops of rat intestine perfused with the amino acids l‐phe, l‐trp, l‐arginine (l‐arg), and l‐glutamine (l‐glut). All four l‐amino acids stimulated a significant rise in GIP and GLP‐1, which was augmented and then blunted by synthetic CaSR agonist and antagonist respectively. Cumulative increases in extracellular calcium concentration have also been found to upregulate l‐phe‐induced GIP and GLP‐1 secretion (Mace et al., 2012). Similar findings have also been observed in pigs by Feng et al. (2019), whereas in humans only GIP release is significantly increased following l‐phe consumption (Amin et al., 2020).

In summary, the CaSR is a promiscuous and pleiotropic amino acid sensor, with a strong affinity for l‐amino acids. Current research has shown that the CaSR directly mediates insulin secretion in human pancreatic islets, however, the presence of extracellular calcium is required for amino acids to fully activate the CaSR. Murine studies have also highlighted that amino acids trigger the secretion of incretins via the CaSR. The role of the CaSR at the level of vagal afferent neurons and in the brain in amino acid sensing, insulin secretion and glucose homeostasis is yet to be ascertained.

#### 3.5.2 The G‐protein‐coupled receptor family C, group 6, subtype A (GPRC6A)

The GPRC6A is a nutrient sensor with a particularly strong affinity for l‐amino acids such as l‐arg, and is highly expressed by pancreatic islets, the brain, the liver, the musculoskeletal system, and the prostate (Christiansen et al., 2007; Pi et al., 2012, 2017; Rueda et al., 2016; Wellendorph et al., 2005). Current evidence for the role of GPRC6A in amino acid‐mediated insulin secretion is somewhat conflicting.

For example, a murine model for global GPRC6A knockout studied by Pi et al. (2012) demonstrated that l‐arg induces insulin secretion in β cells via activation of GPRC6A. Following incubation with l‐arg medium, insulin expression was found to be significantly attenuated in GPRC6A‐deficient islets compared with wild‐type mouse islets ex vivo. This is further supported by Rueda et al. (2016), with l‐ornithine triggering a significant increase in β cell insulin secretion in vitro in murine islets, which was profoundly blunted by introduction of a GPRC6A antagonist.

However, work by Smajilovic et al. (2013) showed that global GPRC6A knockout had no significant effect on l‐arg induced insulin secretion and glycemic control versus wild‐type mice. The discrepancies in the role of GPRC6A in l‐arg induced insulin secretion concluded by Pi et al. (2012) and Smajilovic et al. (2013) may well be due to differences in the knockout model used by each group. This is because Pi et al. (2012) selectively deleted exon 2 of the murine GPRC6A gene to generate GPRC6A knockout mice, whereas Smajilovic et al. (2013) performed deletion of exon 6, which is known to encode the 7‐transmembrane domain and C‐terminal of the GPRC6A gene. The difference in deleted exons may well have created different phenotypes of GPRC6A knockout mice, potentially because exon 2 has a major role in how GPRC6A senses amino acids.

The ability of the GPRC6A to influence peptide hormone release at the level of EECs is also controversial. One study by Oya et al. (2013) supported a role for the GPRC6A in amino acid‐mediated GLP‐1 secretion. Murine EECs incubated with l‐ornithine exhibited a significant rise in intracellular calcium concentration and GLP‐1 secretion, which was significantly depressed following introduction of an established GPRC6A antagonist, versus control vehicle.

In contrast to Oya et al. (2013) and Clemmensen et al. (2016) demonstrated that GLP‐1 secretion may take place independently of the GPRC6A. GPRC6A knockout mice treated with l‐arg and l‐ornithine experienced similar plasma levels of GLP‐1 in wild‐type mice, with no statistically significant difference between the two groups. The reason for the difference in GLP‐1 secretion observed by Oya et al. (2013) versus Clemmensen et al. (2016) could again be explained by contrasting knockout models of GPRC6A. Such that Oya et al. (2013) utilized GLUTag cells with full depletion of GPRC6A by small interfering RNA (siRNA) for GPRC6A, whereas Clemmensen et al. (2016) selectively deleted exon 6 of the GPRC6A gene to achieve knockout. This again questions the role of exon 6 in how GPRC6A senses amino acids.

Overall, the evidence for the role of GPRC6A in amino acid‐dependent insulin and incretin secretion in murine studies is somewhat conflicting, which could be due to phenotypic differences in GPR6CA knockout models. In addition, the roles of GPRC6A with respect to amino acid and protein‐induced insulin and peptide hormone secretion have not yet been reproduced in human islets and EECs, which questions the applicability and validity of the results established in murine studies.

#### 3.5.3 The Umami Taste receptor

The Umami taste receptors are heterodimer G‐protein‐coupled receptors, which contain a common subunit—the T1R3 (Kojima & Nakagawa, 2011). The Umami taste receptor T1R1/T1R3 specifically is a prominent amino acid sensor expressed by pancreatic islets, EECs and glucose‐sensing neurons in the brain (Nakagawa et al., 2009).

Murovets et al. (2015) were one of the first groups to highlight the importance of the Umami T1R1/T1R3 in glucose homeostasis, with a murine global T1R3 knockout model. T1R3 knockout mice exhibited reduced glucose clearance along with diminished insulin sensitivity following glucose tolerance testing, compared to wild‐type mice.

Further research suggests that amino acids could influence insulin secretion via the Umami T1R1/T1R3. Oya et al. (2011) studied insulin secretion from in vitro immortalized murine β‐cells treated with l‐arg or l‐glut in the presence or absence of lactisole, an established antagonist of the murine Umami T1R3 (Hamano et al., 2015). Administration of l‐arg and l‐glut both induced a significant rise in insulin secretion versus unstimulated control β cells, which was significantly blunted when each amino acid was co‐administered with lactisole (Wang et al., 2016).

In comparison with murine studies, there is a paucity of evidence regarding the function of the Umami T1R1/T1R3 in humans with respect to amino acid‐induced insulin or gut hormone secretion. Jang et al. (2007) were one of the few groups which studied the influence of the Umami T1R1/T1R3 in gut hormone secretion in a human cell line. Jang et al. (2007) studied GLP‐1 secretion from the human L‐EEC line NCl‐H716 when incubated with either the artificial sweetener sucralose or a glucose control solution. Sucralose was found to induce a significantly greater increase in GLP‐1 secretion versus glucose, which was blunted by lactisole. Although this study highlights that the Umami T1R1/T1R3 modulates GLP‐1 secretion after sensing glucose and sweeteners, its role in amino acid sensing in human physiology remains unclear.

The Umami T1R1/T1R3 has been shown to influence insulin secretion in murine studies, including l‐amino acid‐mediated insulin secretion. However, the role of the Umami T1R1/T1R3 in humans with respect to amino acid‐mediated insulin and peptide hormone secretion remains far from clear. This must be explored in future research.

#### 3.5.4 The G‐Protein‐Coupled receptor 142 (GPR142)

Like the CaSR and GPRC6A, the GPR142 has a selectively high affinity for l‐amino acids such as l‐trp and l‐phe, and is almost exclusively expressed by pancreatic islets (Rudenko et al., 2019; Wang et al., 2016).

Wang et al. (2016) investigated the ability of GPR142 to modulate insulin secretion by sensing amino acids. In vitro murine islets incubated with l‐trp and a GPR142 agonist (CpdA) respectively both secreted significantly greater insulin levels versus glucose vehicle. Subsequent co‐incubation with an established GPR142 antagonist significantly blunted insulin release with both l‐trp and CpdA. Interestingly, a high glucose concentration was required for significant insulin secretion to be achieved with l‐trp and CpdA. Similar results have also been exhibited with l‐trp by Rudenko et al. (2019), and with Arg and Glut by Capozzi et al. (2019), in which GPR142 activation and in vitro insulin secretion occurred in a glucose‐dependent manner. This could be due to differing roles of glucose and amino acids agonists with respect to GPR142, with glucose potentially as a primary ligand, similar to the role of calcium with respect to the CaSR, whereas amino acids may be acting as positive allosteric modulators of GPR142.

Rudenko et al. (2019) also demonstrated that GPR142 modulates insulin secretion in human islets. Islets treated with the GPR142 agonist C‐I experienced a significant increase in insulin secretion versus control vehicle, with the rate of insulin secretion again occurring in a glucose‐dependent matter as seen in murine islets.

More recent studies by Lin et al. (2016) and Ueda et al. (2018) using global GPR142 knockout mice provide further support for the relevance of GPR142 in amino acid‐mediated insulin secretion. l‐trp was found to significantly stimulate insulin secretion, whilst improving glucose tolerance and glucose‐dependent insulin secretion, which was lost in GPR142 knockout mice.

Despite evidence implicating the GPR142 in amino acid‐induced insulin secretion, the receptor's role in dietary protein‐induced insulin secretion remains less certain. Rudenko et al. (2019) found no significant difference in insulin and glucagon secretion between wild‐type and mice with global GPR142 knockout‐fed whey protein. This could be explained by the less ubiquitous expression of GPR142 throughout the gastrointestinal tract compared with other nutrient sensors like the CaSR, limiting the receptor's contribution to protein‐induced insulin secretion.

Further research into the GPR142 has investigated its influence on EEC function and incretin hormone release. Lin et al. (2016, 2018) demonstrated that treatment with l‐trp and a synthetic GPR142 agonist respectively upregulated GLP‐1 and GIP secretion in both in vitro murine islets as well as in vivo, while also enhancing β‐cell proliferation and giving protection against stress‐induced apoptosis. Significant increases in GLP‐1 secretion following intraduodenal infusion of l‐trp versus control vehicle have also been observed in healthy humans (Steinert et al., 2014).

To summarize current evidence has made clear that l‐amino acids modulate GPR142 to induce insulin and incretin hormone release, which is significantly enhanced in the presence of glucose. It is thus possible that amino acids may act as positive allosteric modulators of GPR142. Like the GPRC6A and the Umami T1R1/T1R3, there is a lack of evidence as to how specific high protein diets modulate insulin secretion and glucose homeostasis via GPR142.

## 4 NON‐INSULINOTROPIC MECHANISMS

The satiating effects of dietary amino acids are also integral to how HPDs may regulate glucose homeostasis (Pal et al., 2014). Dietary amino acids have been shown to suppress appetite and delay return of hunger, resulting in reduced energy intake. In the medium to long term, this can lead to weight loss, improved glucose tolerance and attenuated insulin resistance.

In addition to its insulinotropic abilities, GLP‐1 along with the anorectic hormones cholecystokinin (CCK, secreted by I‐EECs) and peptide tyrosine–tyrosine (PYY, secreted by l‐EECs) regulate food intake (Holst, 2013). Like GLP‐1, CCK and PYY are also secreted in response to dietary amino acid sensing, and all three hormones can inhibit gastric motility and emptying, dampen gastric acid secretion and regulate energy expenditure, which can ultimately contribute to weight loss (Edholm et al., 2010; Seino et al., 2010; Suzuki et al., 2011).

Delayed gastric motility following GLP‐1 and anorectic hormone release have been demonstrated in both rodents and humans (Forster & Dockray, 1992; Ma et al., 2009; Naslund et al., 1998; Tolessa et al., 1998). A whey protein preload 30 min prior to a carbohydrate‐rich meal increased plasma GLP‐1 and CCK levels and delayed gastric emptying preloading in patients with T2DM (Ma et al., 2009). Further work by Gutzwiller et al. (1999) has shown that intravenous infusion of GLP‐1 in T2DM patients significantly reduces food intake and enhances satiety versus placebo control. The same effects have also been observed with PYY, CCK, and pancreatic polypeptide (Batterham, Cohen, et al., 2003; Batterham, Le Roux, et al., 2003; Gutzwiller et al., 2004).

The satiating effects of GLP‐1, pancreatic polypeptide, CCK and PYY have been suggested to be mediated through both peripheral and central pathways (Iwasaki et al., 2017; Krieger et al., 2016; Meryuz et al., 1997; NamKoong et al., 2017; Suzuki et al., 2011). Anorectic hormones are secreted into the gut in the presence of amino acids and fatty acids following meal consumption. Nutrient sensors and chemoreceptors expressed by EECs within the gastric and intestinal mucosa sense amino acids, which subsequently modulates synthesis of specific anorectic hormones within each EEC subtype (Iwasaki et al., 2017; Meryuz et al., 1997; NamKoong et al., 2017). Following synthesis, anorectic hormones are thought to directly modulate afferent and efferent neurons of the vagal and enteric nervous systems which penetrate EECs via neuropods (Bohorquez et al., 2015).

Anorectic hormones can also be secreted by EECs into neighboring capillaries, allowing for absorption into the systemic circulation. Anorectic hormones can subsequently travel to and penetrate the blood brain barrier (Meryuz et al., 1997). This allows anorectic hormones to modulate central receptors expressed by the arcuate nucleus of the hypothalamus, paraventricular nucleus, third ventricle and the dorsal vagal complex of the brainstem. These higher centers ultimately act to bring about satiety.

## 5 CONCLUSION

The consumption of amino acids and HPDs has been shown to mediate insulin secretion through multiple pathways. These mechanisms have been studied in rodents, healthy subjects and patients with T2DM. Nonetheless, there is still a considerable paucity of knowledge as to how nutrient sensors work in order to mediate the effects of amino acids and proteins on glucose homeostasis. Furthermore, it must also be noted that HPDs can be associated with adverse pathophysiological effects, particularly in the long term. Future work should thus focus on exploiting these insulin‐dependent and independent mechanisms without using HPDs, and instead could investigate the effects of specific amino acid supplementation, for example, phenylalanine or arginine in the management of T2DM.

## AUTHOR CONTRIBUTIONS

The entire review article was conceived, written, and edited by the sole author YY.

## FUNDING INFORMATION

This research did not receive any specific grant from any funding agency in the public, commercial, or not‐for‐profit sector.

## CONFLICT OF INTEREST

The author declares that there are no competing interests that could be perceived as prejudicing the impartiality of this review.

## ETHICAL APPROVAL

Not applicable.

## CONSENT TO PARTICIPATE

This is a review article; hence, no human participants were recruited.
